# Editorial: Cytogenomics: Structural Organization and Evolution of Genomes

**DOI:** 10.3389/fgene.2022.938513

**Published:** 2022-06-09

**Authors:** Ricardo Utsunomia, Magdalena Vaio, Francisco J. Ruiz-Ruano

**Affiliations:** ^1^ Department of Genetics, Institute of Biological Sciences and Health, Federal Rural University of Rio de Janeiro, Seropédica, Brazil; ^2^ Faculty of Sciences, São Paulo State University, Bauru, Brazil; ^3^ Department of Plant Biology, Faculty of Agronomy, University of the Republic, Montevideo, Uruguay; ^4^ Department of Organismal Biology—Systematic Biology, Evolutionary Biology Centre, Uppsala University, Uppsala, Sweden; ^5^ School of Biological Sciences, Norwich Research Park University of East Anglia, Norwich, United Kingdom

**Keywords:** repetitive (repeated) DNA, satellite DNA, transposable element (TE), sex chromosomes, supernumerary chromosome, karyotype variability

## Introduction

Cytogenetics is a pioneer field within genetics and emerged when chromosomes were revealed as the gene carriers in the early twentieth century, long before the discovery of the DNA structure. For several decades, cytogenetic studies provided information on the karyotype structure and genome organization of numerous species, revealing a variety of chromosomal polymorphisms in the intra- and interspecies levels.

In the past decade, the power of next-generation sequencing technologies and bioinformatic protocols have become increasingly available to the community of cytogeneticists, allowing the integration of chromosomal and genomic data, even in non-model species of animals and plants, giving rise to the discipline of cytogenomics. The main objective of this Research Topic was to bring together a collection of research and review articles to advance our understanding about karyotype diversification in abroad organism spectrum. In summary, this Topic consists of 11 original papers and one review.

### Repeatomes

Much of the variation in DNA content between eukaryotic species is related to the differential accumulation of a heterogeneous collection of repetitive sequences, defined as the “repeatome”. Here, Pellicer et al. analyzed congeneric species of *Heloniopsis* (Melanthiaceae) that share the same chromosome number, but differ nearly twofold in genome size. Genome skimming data revealed that the differential amplification of existing and distinct LTR-elements and a single satellite DNA are the main drivers of genome amplification in this case. Following this topic, Marino et al. analyzed five already published Cephalopod genomes, which are known to consist in at least 50% of repetitive sequences, to characterize the catalogue of shared repeats between the species. Data revealed the apomorphic nature of retroelement expansion in octopus, while several DNA transposons were found to be conserved in this lineage.

Although phylogenetic analyses are usually based on single-copy genes, the integration of repetitive DNA sequences data can provide some insights into genome evolution, especially in hybrid taxa. In this context, Oliveira et al. performed a comparative analysis of several diploids and allopolyploids *Stylosanthes* species (Leguminosae) with short-read sequencing data. After assembling and characterizing organelle genomes and repeatomes, assembly-free phylogenetic analyses were performed and allowed the recognition of parental genomes in two allopolyplid species, providing a phylogenetic approach for understanding the genome evolution in this group.

### Satellite DNAs

Satellite DNAs (satDNAs) consist of a variety of abundant simple tandemly repeated sequences that are usually located on the peri- and subtelomeric regions. Since its description in the early 60s (Kit 1961), the enzymatic restriction of genomes was the most applied approach to characterize satDNAs. However, this method is not thoroughly efficient, because it is a chance-based approach, since these sequences are highly dynamic and susceptible to quick changes between species, which did not allow a systematic study of multiple satDNAs, even in closely-related species. This scenario changed with the advent and expansion of next generation sequencing technologies and the development of specialized bioinformatic pipelines (Novák et al., 2013), which allowed for a rapid and massive identification of satDNAs from non-model species by taking advantage of low-coverage sequencing.

Conifers are unevenly distributed plant species that exhibit higher abundance on Europe and Asia. Their genomes are largely expanded, mostly exceeding 10 Gb. Here, Heitkam et al. performed a comparative repeat profiling between two conifer species of the *Larix* genus and found that its transposable elements and tandem repeats content were very similar. However, a young satDNA was exclusively found in the European species comparing to the Japanese one, illustrating that the generation of novel repeat families can also play a role in the diversification of conserved conifer genomes.

On the other hand, González et al. analyzed the distribution of four satDNAs in 12 species of *Deschampsia* (Poaceae) and showed that, despite the number of loci, sequence conservation in the monomers of these satDNAs and their chromosomal distribution are quite maintained. Thus, these results suggest that changes in array size and loci number of satDNA, associated with their karyotype and genome diversification, are more marked in these *Deschampsia* species, rather than changes at sequence level.

Since the centromeres are one of the most important structures in cell biology, unveiling their nucleotide composition is a key finding. In this context, Valeri et al. discovered the first centromeric satDNA in two Sirenia species. This satDNA is 684 bp-long and originated after the divergence of Sirenia from Proboscidea and Hyracoidea. Interestingly, no species-specific polymorphisms were found, when comparing several Sirenia species, which is not in accordance with the predictions of concerted evolution and could possibly be related with a centromeric function

### Satellite DNAs and Sex Chromosomes

As largely known, sex chromosomes evolved independently multiple times in the history of life. Once established, a common pattern is observed for these elements and usually includes an intense accumulation of repetitive DNA and heterochromatin, due to its non-recombining nature (Charlesworth et al., 2005). In this context, searching for which repeated DNAs accumulated and where, in the chromosomes, can help us to track the origin and evolution of sex chromosomes in different clades.

Lepidoptera is one of the most diverse groups in nature with the vast majority of species exhibiting an ancestral ZZ/ZW sex chromosome system. Here, Cabral-de-Mello et al. characterized the catalogues of satDNAs in three species within the Crambidae moths and performed comparative analyses between males and females’ genomic libraries for each species. As a whole, they showed a low abundance of satDNAs in this group, but highly differentiated, which was also reflected on the analyzed W chromosomes, that are each following their own evolutionary path.

Contrary to moths, the fish species *Megaleporinus elongatus* exhibit one of the biggest catalogs of satDNAs to date, with 140 different families but comprising only 5% of the genome. Using an integrated approach, Crepaldi et al. tracked the chromosomal clustering of some satDNAs in the sex chromosomes of *M. elongatus* and *M. macrocephalus* and found relevant differences between these species originated recently and that this genome fraction is strongly related to the female sex chromosome differentiation.

### B and Germline-Restricted Chromosomes

Supernumerary, or B, chromosomes are dispensable elements that can perpetuate in natural populations in a parasitic way. Early observations of these elements occurred in the 40s (Östergren 1945) and they were considered inert elements for a long time. However, recent findings, suggest that sometimes B chromosomes can play a significant role by being coopted for essential functions, like sex determination, pathogenicity and others. In this context, Pokorná and Reifová reviewed all such cases of cellular domestication of B chromosomes and showed that, supernumerary elements can be important players with a significant evolutionary impact.

Germline-restricted chromosomes (GRCs) are present in all songbirds studied to date and constitute interesting genomic elements, as they absent in the somatic cells. Torgasheva et al. analyzed the behavior of GRC in male and female meiosis of the great tit and found that GRC was ejected from most male germ cells, corroborating the idea of exclusively maternal inheritance. In addition, chromosome painting analyses revealed that GRCs differ substantially in their genetic content, despite similarities in its behavior during meiosis.

### Molecular Cytogenetics and Karyotype Diversification Patterns

Comparative molecular cytogenetics is still a powerful tool to detect major chromosomal rearrangements. Poignet et al. compared the karyotypes of two passerine species from the genus *Luscinia* by using multiple cytogenetic approaches. Results obtained indicated that diploid chromosome numbers are conserved, as well as main karyotype features, including the presence of similar GRCs. However, comparative genomic hybridization experiments revealed that centromeric repeats in most chromosomes have already diverged, which could theoretically cause meiotic drive or reduced fertility in interspecific hybrids. Following this line, Moraes et al. analyzed five miniature *Pyrrhulina* fishes, which exhibited variation in diploid numbers, as well as differential distribution of repetitive DNAs, suggesting that karyotype diversification in this group has been driven by major structural rearrangements.

## Conclusion and Perspectives

This Research Topic presented studies using a wide variety of approaches and covered several topics in the cytogenetics field. As a whole, this collection demonstrates that the integration of genomic and chromosomal data, and soon, other layers of information, will accelerate our understanding about various aspects of genome evolution.

## Data Availability

The datasets presented in this study can be found in online repositories. The names of the repository/repositories and accession number(s) can be found in the article/Supplementary Material.
